# The Law Relating to Infectious Diseases.—VII

**Published:** 1902-10-04

**Authors:** J. E. R. Stephens

**Affiliations:** Barrister-at-law, Author of "Digest of Public Health Cases," etc. etc.


### The Institutional Workshop.

### THE LAW RELATING TO INFECTIOUS DISEASES.—VII.

By J. E. R. Stephens, Barrister-at-law, Author of "Digest of Public Health Cases," etc. etc.

### Letting House.

Sect. 128 of the Public Health Act, 1875, enacts that any person who knowingly lets for hire any house, room, or part of a house in which any person has been suffering from any dangerous infectious disorder, without having such house, room, or part of a house, and all articles therein liable to retain infection, disinfected to the satisfaction of a legally qualified medical practitioner, as testified by a certificate signed by him, shall be liable to a penalty not exceeding £20. For the purposes of this section, the keeper of an inn shall be deemed to let for hire part of a house to any person admitted as a guest into such inn.

Sect. 129 enacts that any person letting for hire or showing for the purpose of letting for hire any house or part of a house, who on being questioned by any person negotiating for the hire of such house or part of a house as to the fact of there being or within six weeks previously having been therein any person suffering from any dangerous infectious disorder, knowingly makes a false answer to such question, shall be liable, at the discretion of the court, to a penalty not exceeding £20, or to imprisonment, with or without hard labour, for a period not exceeding one month.

In places where the Infectious Disease (Prevention) Act, 1890, has been adopted, persons who occupy a house or part of a house in which there has been a case of infectious disease within the last six weeks before they cease to occupy it, are required to have the premises properly disinfected, and to give notice of the occurrence of the disease to the owner. The outgoing tenant is also liable to a penalty for giving false answers with respect to the occurrence of the disease either to the owner or to a person negotiating for the hire of the premises (53 and 54 Vict., c. 34, s. 7). See also Infectious Disease (Notification) Act, 1889.

### Notification of Infectious Diseases.

The Infectious Disease (Notification) Act, 1889, is only in force where it is adopted, in the manner prescribed by sect. 5. This Act extends to any urban, rural, or port sanitary district after the adoption thereof. Sect. 3 enacts that where an inmate of any building used for human habitation within a district to which this Act extends is suffering from an infectious disease to which this Act applies, then, unless such building is a hospital in which persons suffering from an infectious disease are received, the following provisions shall have effect—that is to say: (*a*) The head of the family to which such inmate (in this Act referred to as the patient) belongs, and in his default the nearest relatives of the patient present in the building or being in attendance on the patient, and in default of such relatives any person in charge of or in attendance on the patient, and in default of any such person the occupier of the building shall, as soon as he becomes aware that the patient is suffering from an infectious disease to which this Act applies, send notice to the medical officer of health of the district; (*b*) every medical practitioner attending on or called in to visit the patient shall forthwith, on becoming aware that the patient is suffering from an infectious disease to which this Act applies, send to the medical officer of health for the district a certificate stating the name of the patient, the situation of the building, and the infectious disease from which, in the opinion of such medical practitioner, the patient is suffering.

Every person required by this section to give a notice or certificate who fails to give the same, shall be liable on summary conviction in the manner provided by the Summary Jurisdiction Acts to a fine not exceeding 40s. Provided that if a person is not required to give notice in the first instance, but only in default of some other person, he shall not be liable to any fine if he satisfies the court that he had reasonable cause to suppose that the notice had been duly given.

When the medical officer of health himself attends the patient he is entitled to the fee under sect. 4 (2) of 2s. 6d. or 1s. as the case may be.

Under sect. 4 the local authority shall gratuitously supply forms of certificate to any medical practitioner residing or practising in their district who applies for the same, and shall pay to every medical practitioner for each certificate duly sent by him in accordance with this Act a fee of 2s. 6d. if the case occurs in his private practice, and of 1s. if the cases occurs in his practice as medical officer of any public body or institution.

In this Act the expression "infectious disease to which this Act applies" means any of the following diseases, namely, small-pox, cholera, diphtheria, membranous croup, erysipelas, the disease known as scarlatina or scarlet fever, and the fevers known by any of the following names: typhus, typhoid, enteric, relapsing, continued or puerperal, and includes as respects any particular district any infectious disease to which this Act has been applied by the local authority of the district.

The local authority of any district to which this Act extends, may, from time to time, by a resolution passed at a meeting of such authority, where the like special notice of the meeting and of the intention to propose the resolution has been given, order that this Act shall apply in their district to any infectious disease other than a disease specifically mentioned in this Act.

The provisions of this Act shall apply to every ship, vessel, boat, tent, van, shed, or similar structure used for human habitation, in like manner as nearly as may be as if it were a building. A ship, vessel, or boat, lying in any river, harbour, or other water not within the district of any local authority, shall be deemed for the purposes of this Act to be within the district of such local authority as may be fixed by the Local Government Board, and where no local authority has been fixed, then of the local authority of the district which nearest adjoins the place where such ship, vessel, or boat is lying. This section shall not apply to any ship, vessel, or boat belonging to any foreign government (sect. 13).

### Schools (Closing of).

In the case of epidemics of certain infectious diseases, *e.g.*, scarlet fever, measles, diphtheria, whooping cough, and small-pox, which spread directly by infection from person to person, the question not infrequently arises whether it is not to the interest of the district that the public elementary schools should be temporarily closed, or that scholars who are likely to bring the disease to the school should be excluded from attendance. The considerations by which a sanitary authority should be guided in giving or refraining from giving a notice to close a school or to exclude certain scholars, are set out very clearly in certain memoranda prepared by the Medical Department of the Local Government Board, which may be studied with advantage by medical officers of health when called upon to advise their sanitary authorities on this subject.

Infection on Ships.

By section 125 of the Public Health Act, 1875, any local authority may make regulations (to be approved of by the Local Government Board) for removing to any hospital to which such authority are entitled to remove patients, and for keeping in such hospital so long as may be necessary any persons brought within their district by any ship or boat who are infected with a dangerous infectious disorder, and such regulations may impose on offenders against the same reasonable penalties not exceeding 40s. for each offence. In 1896 the Local Government Board issued certain regulations relating to infectious diseases breaking out on board ship.

*Concluded.*

